# Clinical significance of pancreatic circulating tumor cells using combined negative enrichment and immunostaining-fluorescence in situ hybridization

**DOI:** 10.1186/s13046-016-0340-0

**Published:** 2016-04-12

**Authors:** Yang Gao, Yayun Zhu, Zhenzhen Zhang, Cheng Zhang, Xinyu Huang, Zhou Yuan

**Affiliations:** Department of General Surgery, Shanghai Jiao Tong University affiliated Sixth People’s Hospital, No.600, Yishan Road, Shanghai, 200233 China; Biotecan Medical Diagnostics Co., Ltd, Zhangjiang Center for Translational Medicine, Shanghai, China

**Keywords:** Circulating tumor cells, Pancreatic cancer, Subtraction enrichment, Diagnosis, Prognosis

## Abstract

**Background:**

Circulating tumor cells (CTCs) hold great potential in both clinical application and basic research for the managements of cancer. However, it remains to be an enormous challenge to obtain efficient detection of pancreatic CTCs. New detection platforms for the detection of pancreatic CTCs are urgently required.

**Methods:**

In the present study, we applied a newly-developed platform integrated subtraction enrichment and immunostaining-fluorescence in situ hybridization (SE-iFISH) to analyze clinical significance of pancreatic CTCs. Immunostaining of CK, CD45, DAPI and FISH with the centromere of chromosome 8 (CEP8) were utilized to identify CTCs. Cells with features of CK+/CD45-/DAPI+/CEP8 = 2, CK+/CD45-/DAPI+/CEP8 > 2, CK-/CD45-/DAPI+/CEP8 > 2 were defined as pancreatic CTCs. The Kaplan-Meier method and Cox proportional hazards model were used to analyze the relationship of CTC level and other clinicopathological factors with pancreatic cancer clinical outcomes.

**Results:**

CTC count in pancreatic cancer was higher than healthy individuals (median, 3 *vs* 0 per 7.5 ml; *P* < 0.001). SE-iFISH platform yielded a sensitivity of 88 % and specificity of 90 % in pancreatic cancer at the cutoff value of 2 cells/7.5 ml. Pancreatic cancer patients with lower CTC count (<3/7.5 ml) had substantially better overall survival (OS) compared with these with higher CTC count (≥3/7.5 ml) (15.2 *vs* 10.2 months, *P* = 0.023). Multivariate analysis indicated that higher CTC count was a strong indicator for worse OS (HR = 4.547, *P* = 0.016).

**Conclusion:**

Our current data showed that CTCs could be detected in pancreatic cancer patients in various stages, whether localized, locally advanced and metastatic. Besides, CTCs have shown the potential implication in predicting prognosis of pancreatic cancer.

## Background

Pancreatic cancer is considered as the one of the most lethal tumor, ranking from eighth and ninth in 2008 in male and female, respectively, to both seventh in 2012 among cancer-related deaths worldwide [1, 2]. In developed countries, pancreatic cancer poses a more severe threat to public health, ranking fifth in man and fourth in women among cancer-related death [2]. In China, pancreatic cancer has been included in the ten most common cancers for male and temporal trend analyses show the incidence rate increased from 2000 to 2011 [3]. Pancreatic ductal adenocarcinoma (PDAC) is the most common and malignant subtype, constituting more than 85 % of all pancreatic malignancy and pancreatic cancer is referred to PDAC in this study [4]. Pancreatic cancer has a dismal 5-year survival rate of approximately 6 % and surgery is the only potentially curative therapy, however, only about 20 % of pancreatic cancers are eligible for resection [5, 6]. The life quality of pancreatic cancer patients is quite poor, which accelerates the cancer-related death [7]. Therefore, early detection and dynamic monitoring of disease progression are crucial for better clinical outcome of pancreatic cancers [6]. Conventional imaging examinations such as contrast enhanced computed tomography (CT) or magnetic resonance imaging (MRI) often fails to detect small primary tumors and small metastasis, optimal treatment opportunities are usually missed [8]. Although carbohydrate antigen 19-9 (CA19-9) is widely used in clinical practice, it is insensitive for early invasive pancreatic cancer and hard to discriminate some pancreatic cancers from benign diseases [6]. Therefore, there is an urgent need for novel biomarkers for early diagnose, dynamical monitoring and risk stratification.

Circulating tumor cells (CTCs) are rare tumor cells disseminated from primary tumors and secondary deposits and act as the seeds of distant metastasis. The concentrations of CTCs in most cancer patients range from 0 to 10 per 10 mL of peripheral blood [9]. In order to improve the efficiency of CTC detection, CTC assays generally include enrichment steps and followed identification procedures [10]. Both physical and biological properties are exploited in the enrichment process. Protein-based strategies and mRNA-based strategies are frequently used in the identification process [11]. CellSearch system is the most intensively tested assay which performs initial enrichment relying on EpCAM (epithelial cellular adhesion molecule) and subsequent identification by cytokeratin (CK) staining of cells in blood samples, but the detection rates are only 20 % and 5-42 % in resectable and advanced pancreatic cancer patients, respectively [12, 13]. Some novel devices should be introduced to promote research and application on pancreatic CTCs.

Recently, a detection platform integrated EpCAM-independent subtraction and immunostaining-fluorescence in situ hybridization (SE-iFISH) has been developed and may facilitate the improvement of pancreatic CTCs [14]. SE-iFISH enables efficient depletion of white blood cells (WBCs) by anti-CD45 antibody and non-hemolytic removal of red blood cells, then in situ phenotypic and karyotypic identification and characterization of centromere probe 8 (CEP8),which could be performed regardless of EpCAM expression and size variations [15]. In this prospective study, SE-iFISH was applied to detect and characterize CTCs in pancreatic cancers and then explored whether CTC number was related to clinicopathological factors and influenced clinical outcomes.

## Methods

### Patients and sample collection

This single-center prospective study was performed in Shanghai Jiao Tong University Affiliated Sixth People’s Hospital. Twenty-five patients with confirmed ductal adenocarcinoma of pancreas (including 5 stage I, 8 stage II, 6 stage III and 6 stage IV) and twenty healthy donors were enrolled from September, 2013 to September, 2015. Written informed consents were obtained from all subjects. Histological examination or radiological imaging analyzed by a multidisciplinary team provided evidence for the diagnosis of PDAC. All patients were subjected to first-line gemcitabine based chemotherapy. Clinical data were collected for age, gender, site of primary tumor, site and number of metastases, tumor size, lymph node metastasis, CA19-9 level and clinical outcomes. TNM staging of pancreatic cancer patients were performed according to American Joint Committee on Cancer (AJCC) 2010 staging.

Peripheral venous blood (7.5 ml) from each subject were collected in customized Acid Citrate Dextrose (ACD)-anticoagulant tubes (Becton Dickinson, NJ, USA) 1 to 10 days before operation and would be processed within 48 h after collection [16]. Consent forms notifying blood samples to be only applied in the present study were signed by all subjects. The study was approved by the Ethics Committee of Shanghai Jiao Tong University Affiliated Sixth People’s Hospital and was performed according to the Declaration of Helsinki Principles.

### Subtraction enrichment

The process for detection of pancreatic CTCs was similar to previous studies [15, 17]. In brief, 7.5 ml peripheral venous blood was centrifuged at 800 g for 8 min at room temperature, then, supernatants above the red blood cells were removed to deplete serum. The left components were mixed with 3 ml hCTC Separation Matrix. The solution was centrifuged at 450 g for 8 min and white buffy was removed to another centrifuge tube, thus depleting red blood cells. The removed white buffy was incubated with 150 μL immunomagnetic particles conjugated to anti-CD45 monoclonal antibody for 10 min and then they were subjected to magnetic separation. Bead-free solution were then transferred to centrifuge tube and washed twice. The resulting cell pellet was mixed with fixative and applied onto coated CTC slides. The slides underwent drying process at 32 °C for 4 h were suitable for iFISH.

### Immunofluorescent staining of CTCs

The identification of CTCs was performed according to kit instruction (Cytelligen). The slides were socked in 2 × SSC at 27 °C for 10 min and dehydrated in 100 % ethanol for 2 min. Centromere Probe 8 (CEP8) SpectrumOrange (Vysis, Abbott Laboratories, Abbott Park, IL, USA) were denatured at 76 °C for 5 min hybridized at 37 °C for 90 min, and the cells were incubated with 200 μl Antibody Preparation Solution-1, 1 μl monoclonal anti-CK18 conjugated to Alexa Fluor 488 and 1 μl monoclonal anti-CD45 conjugated to Alexa Fluor 594 (Cytelligen). The slides were incubated at 30 °C for 2 h in the dark. Finally, sample was washed twice, followed by mounting with 4′,6-diamidino-2-phenylindole (DAPI, Vector laboratories, CA, USA) containing mounting media and subsequently subjected to fluorescence microscope.

### Measurement of CA19-9

Peripheral venous blood (3 ml) was collected into non-anticoagulant blood-collecting tubes (Becton Dickinson, Franklin Lakes, NJ, USA) via centrifugation (1,500 g) at room temperature for 10 min, CA19-9 was measured from the supernatant by using an automatic immunoassay analyzer Cobas e601 (Roche, Pleasanton, CA, USA). The reference range of CA19-9 was less than 37 U/ml.

### Statistical analysis

Statistical analysis was performed with SPSS 17.0 software for windows where a *P* value less than 0.05 was considered statistically significant. The subject’s receiver operating characteristics (ROC) curve was plotted to analyze the sensitivity and specificity of CTCs. Graphpad Prism was used to produce Kaplan-Meier survival curves and log-rank test was used to compare the difference of overall survival (OS). Univariate and multivariate Cox proportional hazards regression analysis were carried out to identify independent risk factor for clinical outcomes [18]. All the statistical tests were two-sided.

## Results

### Identification of CTCs from pancreatic cancer patients

**Fig. 1:**
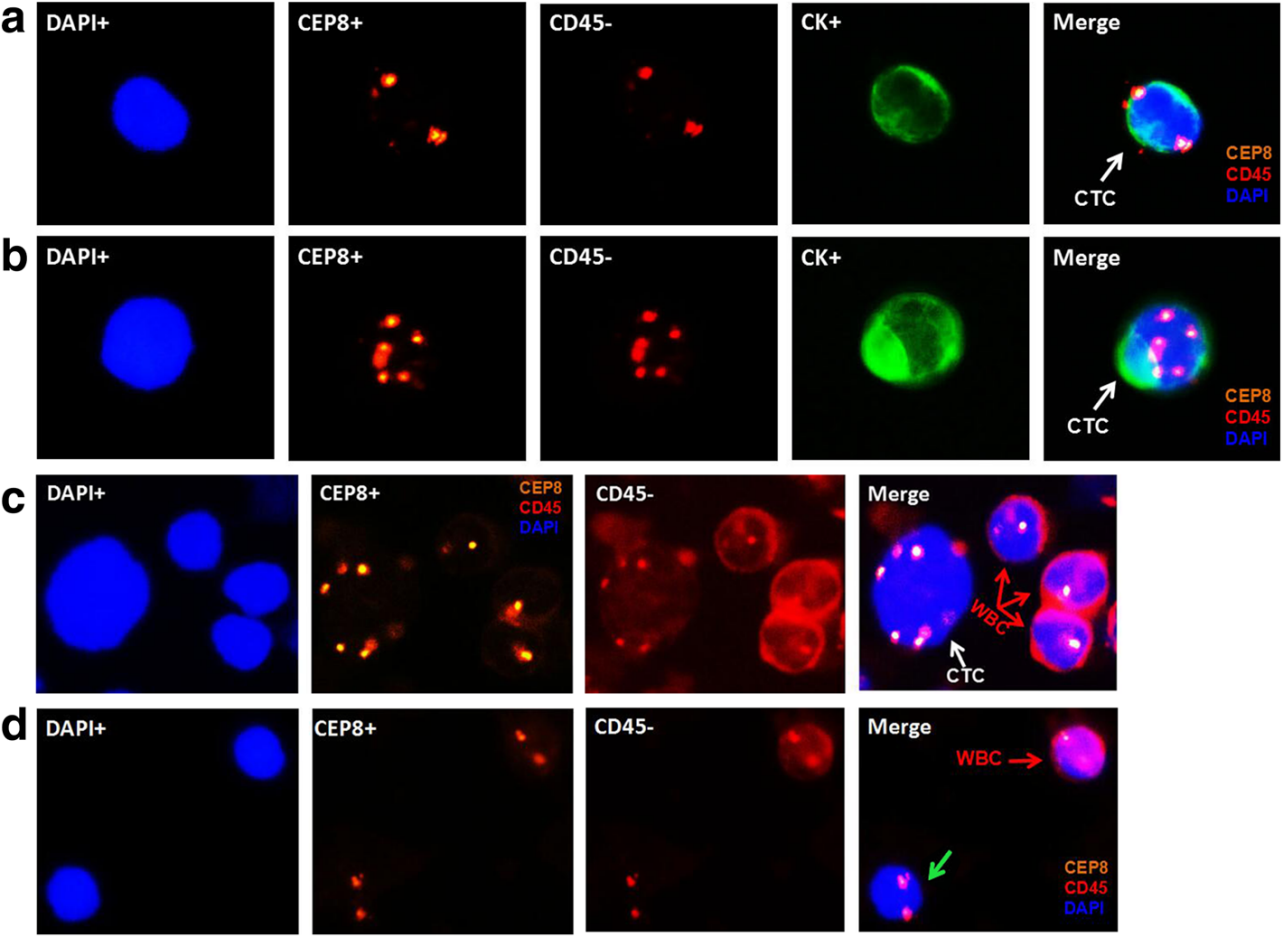
Identification of CTCs in pancreatic cancer by SE-iFISH platform. CK: green, CEP8: orange, DAPI: blue, CD45: red. **a** CK+/CD45-/DAPI+/CEP8 = 2 (white arrow); **b** CK+/CD45-/DAPI+/CEP8 > 2 (white arrow); **c** CK-/CD45+/DAPI+/CEP8 > 2 (white arrow); **d** CK-/CD45+/DAPI+/CEP8 = 2 (red arrow), CK-/CD45-/DAPI+/CEP8 = 2 (green arrow)

Various kinds of cells were differentiated by epithelial marker (CK), hematopoietic WBC marker (CD45), existence of cell nucleus (DAPI) and chromosome ploidy (CEP8). These biomarkers had been frequently applied in previous studies in some cancer types [17, 19]. Therefore, we were inspired by previous studies and adopted these markers to identify CTCs in pancreatic cancers. In general, CTCs were characterized as nucleated cells with epithelial markers and/or hyperdiploid but without CD45 (Fig. 1). To be specific, CTCs were defined as CK+/CD45-/DAPI+/CEP8 = 2, CK+/CD45-/DAPI+/CEP8 > 2, CK-/CD45-/DAPI+/CEP8 > 2. CK-/CD45+/DAPI+/CEP8 = 2 was defined as WBC and CK-/CD45-/DAPI+/CEP8 = 2 were defined as indeterminate cells.

According to the above definition, a total of 103 CTCs were detected among the 25 pancreatic cancer patients. The features of most CTCs were CK-/CD45-/DAPI+/CEP8 > 2, which appeared in 24 of the 25 pancreatic cancer patients and accounted for 96.1 % of the whole CTCs. Only 1 was CK+/CD45-/DAPI+/CEP8 = 2 in 1 pancreatic cancer patient and 3 were CK+/CD45-/DAPI+/CEP8 > 2 in 2 pancreatic cancer patients. In addition, 6 CTCs in 4 healthy controls were CK-/CD45-/DAPI+/CEP8 > 2 and positive CK was not detected among them. The indeterminate cells were quite common. Among pancreatic cancer patients, indeterminate cells appeared in 21 of the 25 subjects with a median number of 4 CTCs/7.5 ml (range, 0-8 CTCs/7.5 ml). Among healthy controls, indeterminate cells appeared in 13 of the 20 subjects with a median number of 1 CTCs/7.5 ml (range, 0-5 CTCs/7.5 ml).

### CTCs in pancreatic cancer patients and healthy controls

**Fig. 2:**
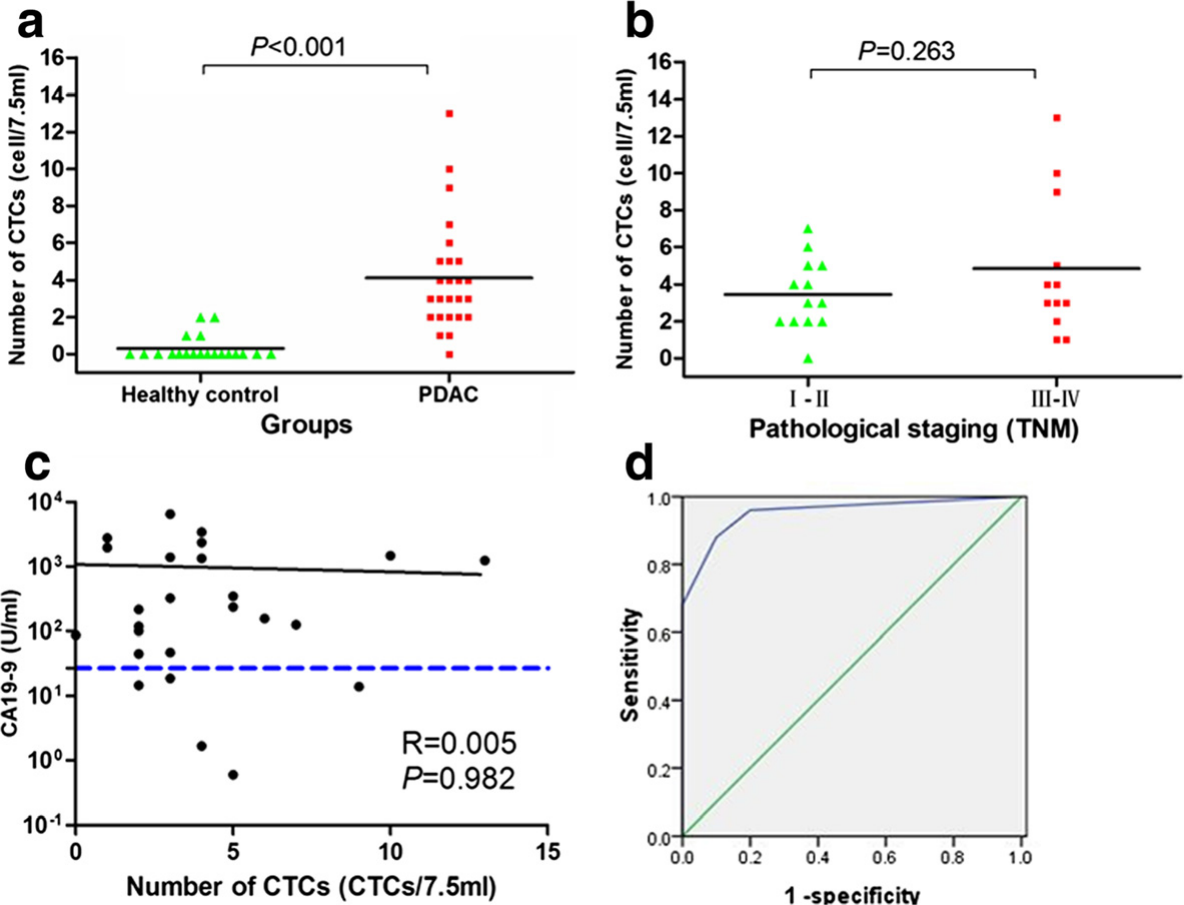
CTC count in pancreatic cancer patients and healthy controls. **a**, Distribution of CTCs in diagnosed pancreatic cancer and health controls, existence of CTCs in 25 pancreatic cancer patients and 20 healthy controls were examined. **b** Distribution of CTCs in patients with pancreatic cancer according to pathological staging (TNM). **c** Comparison of circulating tumor cells and CA19-9 as blood-based markers. The blue horizontal dashed line indicated the CA 19-9 threshold of 37 U/ml. **d** ROC curves for CTCs count to discriminate pancreatic cancer patients from healthy controls. The cutoff value was defined as 2 CTCs/7.5 ml

According to above definition, CTC number was 0-13 CTCs/7.5 ml (median number, 3 CTCs/7.5 ml) in pancreatic cancer patients and 0-2 CTCs/7.5 ml (median number, 0 CTCs/7.5 ml) in health donors. These difference were statistically significant (*P* < 0.001) (Fig. 2a). CA19-9 and TNM stage were both important clinicopathological factors for pancreatic cancer, therefore we compared the relationship between these two factors and CTC enumeration. The median CTC number per 7.5 ml peripheral blood in stage I-II and stage III-IV were 3 (range, 0-7) and 3.5 (range, 2-13), respectively, no significant difference were found between them (*P* = 0.263) (Fig. 2b). The median CA19-9 level was 216 U/ml (range, 0.6-6540 U/ml) and CA19-9 levels were elevated (>37 U/ml) in 20 of the 25 pancreatic cancer patients (80 %). The Spearman correlation (R) between CA19-9 levels and CTC levels was 0.005 (*P* = 0.982), so CA19-9 and CTC were relatively independent parameters (Fig. 2c). Furthermore, CTC enumeration was not associated with gender, age, tumor size, lymph node metastasis, and distant metastasis.

In order to discriminate pancreatic cancer patients from healthy controls, receiver operating characteristic (ROC) curve was plotted to determine sensitivity and specificity of CTCs measured by SE-iFISH platform (Fig. 2d). According to Yourdon’s index, cutoff values of 1 CTC/7.5 ml, 2 CTCs/7.5 ml, 3 CTCs/7.5 ml, and 5 CTCs/7.5 ml yielded sensitivities of 96 %, 88 %, 68 %, 32 % and specificities of 80 %, 90 %, 100 %, 100 %. Therefore, we defined cutoff as 2 CTCs/7.5 ml (AUC = 0.954) and a CTC-positive patient was defined as one whose CTC count in 7.5 mL peripheral venous blood was ≥2. The positive rate of CTCs in stage I–II and stage III-IV were 92.3 % (12/13) and 83.3 % (10/12), with no significant difference between them (*P* = 0.593). Similarly, CTC-positive rates among patients with lower CA19-9 level (<200 U/ml) were not significantly different with these with higher CA19-9 level (≥200 U/ml) (*P* = 1.000). Noticeably, 5 patients with normal CA19-9 levels were all CTC-positive patients, so the detection rate of pancreatic cancer could reach 100 % when CTC detection and CA19-9 detection were combined. In addition, CTC positive rates were not significantly associated with gender, age, tumor size, lymph node metastasis, and distant metastasis (Table 1).

**Table 1:** The relationship between CTC-positive rate and clinicopathological characteristics

Variations	Number (%)	No of patients		*P*
		CTC-positive CTC-negative		
Gender				
Male	16	13	3	0.280
Female	9	9	0	
Age				
<60	8	7	1	1.000
≥60	17	15	2	
Tumor stage				
I-II	13	12	1	0.593
III-IV	12	10	2	
Tumor size				
T1-T2	9	8	1	1.000
T3-T4	16	14	2	
Lymph nodes				
N0	8	8	0	0.527
N1	17	14	3	
Metastasis				
M0	19	18	1	0.133
M1	6	4	2	
CA19-9				
<200 U/ml	12	11	1	1.000
≥200 U/ml	13	11	2	

### Survival analysis

**Fig. 3:**
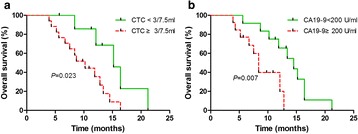
OS in patients different level of CTCs and CA19-9. **a** OS in patients with lower CTC count (CTC < 3/7.5 ml ) had better OS compared these with higher CTC count (CTC ≥ 3/7.5 ml) (15.2 *vs* 10.2 months, *P* = 0.023); **b** OS in patients with lower CA19-9 level (CA19-9 < 200 U/ml) was superior to these with higher CA19-9 level (CA19-9 ≥ 200 U/ml) (14.5 *vs* 8.4 months, *P* = 0.007)

During the diligent follow-up of 24 months, the median follow-up time was 10.2 months (range, 3.9-21.1 months) for deceased patients and 10.3 months (range, 7.5–11.3 months) for living patients. The median OS of the 25 pancreatic cancer patients was 10.2 months (range, 3.9-21.6 months). Patients in stage I-II had a significantly longer median OS compared to these in stage III-IV (13.4 *vs* 7.5 months, *P* = 0.001). To evaluate the influence of the cutoff of CTC count and CA9-9 level on the hazard ratios of OS, the CTC number of 2, 3, 5 per 7.5 ml and CA19-9 value of 37, 200, 1000 per ml were tested for correlation with OS by Kaplan-Meier method. Finally, 3 CTCs/7.5 ml and 200 U/ml were chosen to analyze the prognostic significance of CA19-9 and CTCs, respectively. The median OS in patients with CTC < 3/7.5 ml was significantly superior to these with CTC ≥ 3/7.5 ml (15.2 *vs* 10.2 months, *P* = 0.023) (Fig. 3a). Similarly, the OS was favorable in patients with a level of CA19-9 < 200 U/ml compared to these with a value above (14.5 *vs* 8.4 months, *P* = 0.007) (Fig. 3b). We also strived to explore the correlation between OS and multiploidy of chromosome 8 (containing pentaploidy and those > 5 copies), but failed to find the prognostic significance (*P* = 0.119).

CTC enumeration and other clinical factors, including sex, age, TNM staging, and CA19-9 level were subjected univariate Cox proportional hazards regression analysis to determine if they were correlated to OS. Only the clinical factors that were significantly associated with OS by the univariate analysis were included in the multivariate Cox regression analyses (Table 2). As a result, CTC count, TNM staging, CA19-9 level, and age were qualified for further analysis. In the multivariate Cox proportional hazards regression analysis, CTC count remained to be a strong predictor of OS (HR, 4.547; 95 % CI: 1.323–15.625; *P* = 0.016).

**Table 2:** Univariate and multivariate Cox proportional hazards regression analysis for prediction of OS

Parameter	*P* value	HR	95 % CI
Univariate Cox proportional hazards regression analysis			
CTC ≥ 3/7.5 ml	0.037	3.383	1.079–10.608
Higher TNM staging	0.014	4.689	1.374–16.000
CA19-9 ≥ 200 U/ml	<0.001	3.216	1.674–6.192
Age ≥ 60	0.040	3.853	1.063–13.967
Multivariate Cox proportional hazards regression analysis			
CTC ≥ 3/7.5 ml	0.016	4.547	1.323–15.625
Higher TNM staging	0.012	2.742	1.245–6.041
CA19-9 ≥ 200 U/ml	0.248	2.332	0.555–9.796
Age ≥ 60	0.366	1.990	0.447–8.863

Note: Cox proportional hazards models. All statistical tests were two-sided. CTCs were collected in 7.5 ml peripheral venous blood. OS, overall survival; CTC, circulating tumor cell; HR, hazard ratio; CI, confidence interval

## Discussion

Circulating tumor cells have attracted more and more attention in both cancer research and clinical practice as a useful surrogate biomarker. It has been reported that CTCs could be detected in all stages of pancreatic cancer and even pre-cancerous disease [20–22], therefore, CTCs could be regarded as the source of metastasis. Because most imaging modalities failed to detected pancreatic lesions smaller than 1 cm, CTCs are promising to detect the occult primary or metastatic tumors [23]. CTCs could predict an unfavorable prognosis, which may influence the treatment decision for better management of pancreatic cancer in clinic [12, 13]. What’s more, CTCs cultured in vivo and vitro have potential implications for drug sensitivity screening, metastatic mechanism and drug resistance, which will promote further study on pancreatic cancer [24, 25].

In this study, SE-iFISH was applied to detect CTCs in pancreatic cancer of various stages. This distinguished subtraction enrichment took advantage of anti-WBC markers antibodies to ensure the depletion of WBCs (as high as 99.99 %) and minimum hypotonic injury to CTCs [14, 26]. Conventional EpCAM-dependent enrichment methods have some inherent limitations which hindered the efficient detection of pancreatic CTCs. One the one hand, EpCAM was expressed only in about 20 % of pancreatic patients through immunomagnetic enrichment by using the antibodies targeting mucin 1 and EpCAM [27]. On the other hand, anti-EpCAM antibody could trigger intracellular signaling pathways, such as cell adhesion, proliferation, which had an impact on the following functional analysis [28, 29]. The modified enrichment method yields CTCs regardless of the EpCAM expression status and cell size.

In the identification process, CEP8, CK, CD45 and DAPI were combined to detect CTCs. Since aneuploidy is a typical common cytogenetic abnormality malignant cells, this feature could be exploited for CTC detection [30]. Previous studies have revealed that centrosome abnormalities appeared in 85 % of pancreatic cancers and centrosome abnormalities of chromosome 8 appeared in all of 16 analyzed pancreatic cell lines using FISH, thus, the variation of chromosome numbers could be reflected by CEP8 [31, 32]. In our series, almost all CTCs harbored multiple CEP8 (≥3). Aneuploidy of chromosome 8 examined by CEP8-FISH also occurred in other cancer types, such as lung, esophageal, gastric, colon, bladder, and hepatocellular carcinomas and so on [15]. The aneuploidy of chromosome could affect the sensitivity of chemotherapy. Yilin Li et al. reported that the primary chemoresistance of triploid CTCs was intrinsic and chemoresistance for tetraploid and multiploid CTCs were acquired in gastric cancer, which cast light on the functional analysis of aneuploidy of chromosome 8 [17]. In addition, CEP8 combined with CEP7 have also been utilized to identify in lung cancer and increased the detection rate compared to CEP8 alone or CK-based method [19, 33]. For these cancer types with low detection rate with single centrosome probe, multiple probes are feasible to improve sensitivity.

Cytokeratins are most frequently used epithelial markers but could be down-regulated and even missed in the epithelial to mesenchymal transition (EMT) process [34–36]. This theory could account for the fact that most CTCs detected by SE-iFISH were usually CK-negative [15, 37, 38]. Having analyzed the significance of subtraction enrichment, CEP8 and CK, the definition of CTCs could be further understood. Cells with characteristics of CK+/CD45-/DAPI+/CEP8 = 2, CK+/CD45-/DAPI+/CEP8 > 2, CK-/CD45-/DAPI+/CEP8 > 2 were CTCs because of positive epithelial and/or hyperdiploid. Cells with characteristics of CK-/CD45+/DAPI+/CEP8 = 2 were WBCs because CD45 is leukocyte-specific transmembrane protein tyrosine phosphatase and diploid of chromosome 8 [39, 40]. Cells with features of CK-/CD45-/DAPI+/CEP8 = 2 were indeterminate cells, which could be either WBCs with unstained human anti-CD45 antibody or tumor cells with negative CK. However, the clinical significance of these indeterminate cells was still unclear.

In the present study, SE-iFISH achieved a sensitivity of 88 % and specificity of 90 % at the cutoff value of 2 CTCs/7.5 ml in pancreatic cancer. CTC detection rates were independent of some conventional clinicopathological factors, such as TNM staging, CA19-9 level, gender and sex. However, the relationship between CTC amount and age, gender, tumor size, CA19-9 may be false negative since this conclusion was drawn from a small cohort only including 25 pancreatic cancer cases. So we should pay attention to this point and make proper explanation on this conclusion. Specially, CTCs could be detected in early-stage pancreatic cancer and this may promote the early diagnose of pancreatic cancer [41]. CTCs are most useful at the early stage of pancreatic cancer because there are multiple treatment options to prolong the survival and the clinical outcomes of late-stage pancreatic cancer are dismal despite exhausted managements. CA19-9 is the most commonly used tumor marker in monitoring response to treatment for pancreatic cancer but was not recommended as a screening test due to poor and variable sensitivity (70–90 %) and specificity (68–91 %) [6]. Besides, about 5 % of population was Lewis^a-b-^ and no elevation in CA19-9 levels will occurred in them [42]. Combination of CTC and CA19-9 detection will significantly increase the detection pancreatic cancer. In this study, the detection rate of pancreatic cancer reached 100 % by combing CTC and CA19-9. From the standpoint of diagnose, CTC number was a complementary parameter of CA19-9 level in diagnosis of pancreatic cancer. Therefore, CTCs and CA19-9 may work as a useful screening tool for individuals with high-risk for pancreatic cancer [21].

The prognostic significance of CTCs in pancreatic cancer is still controversial. It seems that the conclusion was related to diversified detection platform, such as CellSearch system, size-based CTC and SE-iFISH. Bidard et al. reported that 9 out of 79 patients (11 %) have one or more CTCs by CellSearch system, and the difference in OS was found (RR = 2.5, *P* = 0.01) but progress free survival wasn’t found (*P* = 0.24) between CTC-positive patients and CTC-negative patients [13]. Due to the small number and low detection rate, the statistical power to confirm the conclusion is not enough. Recently, an Italian study team adopting the same method failed to found significance in both overall and disease-free survival between CTC-positive and CTC-negative patients, but they found CTCs appeared in portal vein predicated liver metastases [12]. The low number of CTCs detected by CellSearch system may stem from low or negative expression of EpCAM and/or CKs [43]. Since the EpCAM-dependent device couldn’t produce satisfactory results, EpCAM-independent device have been applied to pancreatic CTCs. Size-based devices were used to detect CTCs more accurately and efficiently. However, different modification of sized-based device also yielded different conclusions. L Khoja et al. isolated blood samples via isolation by size of epithelial tumor cells (ISET) and then identify them solely by morphological characters, such as nuclear to cytoplasmic ratio, diameter, hyperchromatic nuclei and cellular shape [43]. Although they detected CTCs in 93 % of pancreatic cancer patients, they didn’t find significant difference in both progress free survival (*P* = 0.85) and OS (*P* = 0.36) [43]. Birte Kulemann et al. adopted another size-based device, ScreenCell, to enrich CTCs and then identify them by morphological features. Similarly, they also failed to reveal the prognostic significance of CTCs [34]. Recently, a study took advantage of ISET device and CTCs were defined according to cell size, and expression of cytokeratin (epithelial marker), vimentin (mesenchymal marker), CD45 [22]. Then, cytokeratin-positive CTCs proved to be an adverse factor for OS successfully (*P* = 0.008). This success contributed the application of molecular biomarker and further revealed the underlying factors for prognosis. Our study confirmed that higher level of CTCs detected by SE-iFISH method was associated with adverse OS (*P* = 0.023, HR = 4.547) [37]. CTC level, together with TNM stage and CA19-9 level could predict the survival of pancreatic cancer. Pancreatic cancer patients with high level of CTCs tend to have worse prognosis and efficient managements, including accurate diagnose, optimal surgical scheme and medical treatment should be assigned to improve clinical outcomes.

Although this study was a prospect study, there were several limitations. Firstly, the sample size was still quite small and the number of patients in various pathological stages was low. So, they were roughly divided into two subgroups to analyze the difference of CTC number and OS. Still, the difference of OS between patients in stage I–II and stage III-IV was statistically significant. Secondly, the dynamic monitoring of CTCs was not performed in this study due to high cost of detection. Previous studies have reported CTC enumeration change during perioperative period in a fraction of patients, however, CTC enumeration after several cycles of chemotherapy and the significance of CTC change still required further study [19, 37]. Thirdly, we presented the AUC in the cross-sectional analysis and the cutoffs of CTCs and CA19-9 in the prospective analysis. These results might be applicable for pancreatic cancer patients with similar characteristics including clinical stages of cancer. However, the external validity of the results was unknown. Therefore, more verification experiments are expected to further confirm the present conclusion and these future researches will be conductive to promoting the clinical application of CTC detection.

## Conclusions

In summary, SE-iFISH platform could detect CTCs in pancreatic cancer of various pathological stages. The sensitivity and specificity were 88 % and 90 % at the cutoff value of 2 cells/7.5 ml. The detection rate of pancreatic cancer could be 100 % when CTC level and CA19-9 level were combined. What’s more, higher level of CTCs successfully predicted unfavorable prognosis and this parameter will inform the clinicians to pay special attention to CTC-positive patients.

## Abbreviations

AJCC: American joint committee on cancer
CA19-9: carbohydrate antigen 19-9
CEP8: centromere of chromosome 8
CK: cytokeratin
CTC: circulating tumor cell
DAPI: 4′,6-diamidino-2-phenylindole
EpCAM: epithelial cellular adhesion molecule
FISH: fluorescence in situ hybridization
HR: hazard ratio
OS: overall survival
ROC: receiver operating characteristics
SE-iFISH: subtraction enrichment and immunostaining-fluorescence in situ hybridization
WBC: white blood cell
